# Multistate Models Reveal Long-Term Trends of Northern Spotted Owls in the Absence of a Novel Competitor

**DOI:** 10.1371/journal.pone.0152888

**Published:** 2016-04-11

**Authors:** Andrew J. Kroll, Jay E. Jones, Angela B. Stringer, Douglas J. Meekins

**Affiliations:** 1 Weyerhaeuser, Federal Way, Washington, United States of America; 2 Fire Mountain Consulting LLC, Vancouver, Washington, United States of America; 3 Campbell Global LLC, Cathlamet, Washington, United States of America; University of Missouri Kansas City, UNITED STATES

## Abstract

Quantifying spatial and temporal variability in population trends is a critical aspect of successful management of imperiled species. We evaluated territory occupancy dynamics of northern spotted owls (*Strix occidentalis caurina*), California, USA, 1990–2014. The study area possessed two unique aspects. First, timber management has occurred for over 100 years, resulting in dramatically different forest successional and structural conditions compared to other areas. Second, the barred owl (*Strix varia*), an exotic congener known to exert significant negative effects on spotted owls, has not colonized the study area. We used a Bayesian dynamic multistate model to evaluate if territory occupancy of reproductive spotted owls has declined as in other study areas. The state-space approach for dynamic multistate modeling imputes the number of territories for each nesting state and allows for the estimation of longer-term trends in occupied or reproductive territories from longitudinal studies. The multistate approach accounts for different detection probabilities by nesting state (to account for either inherent differences in detection or for the use of different survey methods for different occupancy states) and reduces bias in state assignment. Estimated linear trends in the number of reproductive territories suggested an average loss of approximately one half territory per year (-0.55, 90% CRI: -0.76, -0.33), in one management block and a loss of 0.15 per year (-0.15, 90% CRI: -0.24, -0.07), in another management block during the 25 year observation period. Estimated trends in the third management block were also negative, but substantial uncertainty existed in the estimate (-0.09, 90% CRI: -0.35, 0.17). Our results indicate that the number of territories occupied by northern spotted owl pairs remained relatively constant over a 25 year period (-0.07, 90% CRI: -0.20, 0.05; -0.01, 90% CRI: -0.19, 0.16; -0.16, 90% CRI: -0.40, 0.06). However, we cannot exclude small-to-moderate declines or increases in paired territory numbers due to uncertainty in our estimates. Collectively, we conclude spotted owl pair populations on this landscape managed for commercial timber production appear to be more stable and do not show sharp year-over-year declines seen in both managed and unmanaged landscapes with substantial barred owl colonization and persistence. Continued monitoring of reproductive territories can determine whether recent declines continue or whether trends reverse as they have on four previous occasions. Experimental investigations to evaluate changes to spotted owl occupancy dynamics when barred owl populations are reduced or removed entirely can confirm the generality of this conclusion.

## Introduction

Long-term ecological studies often investigate population dynamics as a function of habitat quality, competition, meta-population structure, and other factors. Collection of demographic data such as fecundity and survival can be challenging and costly, however, and limit their application in many instances. For long-lived species that exhibit strong site fidelity, evaluation of multiple occupancy states may be an effective alternative to support conservation and management programs. Collection of detection/non-detection data is relatively simple, and a broad array of sampling designs and statistical tools are available to analyze basic and applied questions [1, 2]. For species of particular conservation interest, utilizing these tools across multiple studies can provide strong inference about factors affecting population dynamics.

The northern spotted owl (*Strix occidentalis caurina*) is an endangered raptor which exhibits a strong association with structurally complex conifer forests in the Pacific Northwest, USA [3, 4]. The current endangered status of the spotted owl [5] results from population declines associated with reductions of preferred late-successional forest habitat due to timber harvesting. Management agencies proposed broad-scale conservation regulations under the assumption of continued declines in spotted owl populations until improved habitat conditions could support increasing populations [4]. However, recent, rapid expansion of barred owl (*Strix varia*) populations throughout much of the distribution of the spotted owl exacerbated population declines [6–9]. Although presence of this novel ecological competitor can have negative consequences for spotted owl productivity and adult survival [10, 11], Anthony et al. [10] found little support for a negative association between barred owl presence and spotted owl fecundity. Instead, barred owl presence may exert a negative effect on spotted owl territory occupancy [9, 10]. For example, recent analyses found strong associative evidence for interference competition between the two species and consequent negative effects on spotted owl territory occupancy [12, 13]. However, due to the near ubiquity of barred owls throughout the distribution of spotted owls, information is not available to evaluate the original premise that spotted owl populations would recover as habitat conditions improved and as conservation measures were implemented where active timber management continued to occur [4].

In this paper, we used a multistate occupancy model to evaluate northern spotted owl territory occupancy and reproductive dynamics in northern California, USA, 1990–2014. Our primary question was whether territory occupancy of reproducing spotted owls has declined over time, as in other populations of this species [11, 14, 15]? Our dataset and analysis are of broad interest for two reasons. First, we sampled territories in a landscape managed for timber production throughout the 25 year period of observation. Second, this portion of the spotted owl’s distribution does not support breeding populations of the barred owl. Current investigations in other areas will evaluate if spotted owl occupancy dynamics change once barred owl populations are reduced or removed entirely [16]. As a result, our analysis provides unique insight into contemporary population dynamics of the spotted owl, and can complement on-going studies to inform management activities to conserve spotted owl populations.

## Materials and Methods

### Study Area & Management Descriptions

Our study area was located in Mendocino County, CA, USA (S1 Fig). Three generally contiguous blocks occurred from north to south: Blocks A (209 km^2^), B (472 km^2^), and C (107 km^2^). Elevations ranged from 0‒915 m (3000 feet). The furthest inland extent of the study area was 33 km. The climate is characterized by cool, wet winters and hot, dry summers [17]. Fog is the most common source of precipitation during the summer months, particularly along the coast and in coastal valleys [17]. The study area contained a mix of Redwood, Douglas-fir, Montane Hardwood, and Montane Hardwood-Conifer forest vegetation types [18]. Dominant tree species included coast redwood (*Sequoia sempervirens*), as well as Douglas-fir (*Pseudotsuga menziesii*), grand fir (*Abies grandis*), and western hemlock (*Tsuga heterophylla*). Tanoak (*Notholithocarpus densiflorus*), Pacific madrone (*Arbutus menziesii*), and red alder (*Alnus rubra*) were the most common hardwood species, with tanoak and madrone dominant on xeric and higher elevation sites. Common understory shrub species included ceanothus (*Ceanothus* spp.), coyotebush (*Baccharis pilularis*), huckleberry (*Vaccinium* spp.), manzanita (*Arctostaphylos* spp.), poison-oak (*Toxicodendron diversilobum*), rhododendron (*Rhododendron macrophyllum*), and salal (*Gaultheria shallon*).

Commercial timber management has occurred in the study area for more than a century and all of the forest stands were either 2^nd^ or 3^rd^-growth with most stands less than 80 years old. Numerous timber management techniques have been applied over the decades, including both even- and uneven-aged silvicultural prescriptions. As a result, the study area contained a mosaic of forest stands relative to species composition, stand structure, and age distribution. Late-successional forest structures occurred on the blocks only in the form of individual trees or clumps of residual trees.

### Northern Spotted Owl Surveys & Territory Monitoring

The spotted owl population in this study area has been monitored since the late 1980s when public and private landowners began implementing standardized survey protocols to determine spotted owl presence and population status on their respective ownerships [19, 20]. In order to maintain compliance with state and federal “take avoidance” requirements, spotted owl surveys in this study area have followed the survey protocol current at the time [20–22]. When spotted owls were detected during surveys, we initiated follow-up monitoring [20] and applied standard protection measures for the spotted owl territory.

In general, we conducted spotted owl surveys between March 1^st^ and August 31^st^ across all three blocks. The exact count of survey visits to a single territory varied each year due to the timing and type of spotted owl detection, evidence of breeding effort, and proximity to timber management activities. We used one of two methodologies for each spotted owl survey visit: nighttime point-calling surveys in areas without known spotted owl territories and daytime walk-in surveys for known territories. In this analysis, we used data only from daytime walk-in surveys. As a result, our sample includes territories known to be occupied prior to 1990 as well as territories identified (during nighttime surveys) after 1990 with unknown status prior to 1990.

We used daytime surveys to determine occupancy status (i.e., single or pair) and reproductive status. Here, we define positive reproductive status as an owl pair provisioning nestlings, consistent with prior usage [23]. Daytime surveys were conducted in the historic territory site center(s) or in areas where spotted owls were detected during nighttime surveys. We conducted daytime surveys approximately two hours before sunset. Daytime surveys consisted of using spotted owl vocalizations to elicit an owl response and searching for evidence (i.e., pellets and white-wash) of owl presence.

We fed live mice to spotted owls located during daytime surveys to determine territory status and nesting activity. For surveys conducted before May 15^th^, if an owl cached or ate four mice on a minimum of two visits it was considered to be ‘non-nesting’; if an owl took a mouse to a nest it was considered to be ‘nesting’. We suspended surveys for non-nesting owls after May 30^th^, with the exception of banding visits for un-banded individuals. We concluded young were present when owls took mice to the nest (reproductive status was positive).

Our dataset consisted of 104 territories monitored from 1990–2014. Eighteen, 62, and 24 territories occurred on Blocks A, B, and C, respectively. Although surveys occurred prior to 1990, we did not include those data because the survey program was still being developed and the study area had not been surveyed thoroughly. In any given year, we surveyed all known and active spotted owl territories. We discontinued surveys at previously active territories only after completing absence surveys under the USFWS protocol [21] or declared abandoned in consultation with USFWS. At the same time, we added new territories to the survey program once identified. An individual territory was considered eligible for inclusion in the database if it had at least one survey in a season, regardless of whether or not an owl was detected. We note that 73/104 (70%) of the territories were identified and surveyed in the first 3 years of the study (1990–1992). We surveyed an average of 67 territories (SD = 16; range = 36–94) with daytime visits in each year. Individual territories were surveyed from 1–15 times in each year (median = 3 surveys per year; average = 3.5; SD = 1.8), with a median range of 71 days between first and last visits (minimum = 0, maximum = 150). On Block C, we discontinued surveys after 2006. We recorded 25 barred owl detections on 11 territories (11%) during the duration of the study. Number of detections on these 11 territories ranged from 1–5. We detected seven barred owls from 1990–1992 and five barred owls from 2012–2014.

### Analytical Approach for Estimating Multistate Occupancy Dynamics

We used the MacKenzie et al. [23] dynamic multistate occupancy model to examine trends in NSO occupancy states. This methodology allowed for estimation of state probabilities and across-year state transition probabilities, while accounting for imperfect state detection. In our analysis, we considered four possible states: unoccupied (state 1), occupied by single owl (state 2), occupied by non-reproducing pair (state 3), and occupied by reproducing pair (state 4).

We followed the state-space approach described in MacKenzie et al. [23], where the true state of a territory is taken to be a random vector **z**. For example, if the true state of territory *i* at time *t* was occupancy by a non-reproducing pair (state 3), this would give **z**_*it*_ = [0 0 1 0]. The random vector **z** is assumed to arise from a single draw of a multinomial distribution. The initial state probabilities are denoted $\phi_{0}=\left[\phi_{0}^{\;\left[1\right]}\;\phi_{0}^{\;\left[2\right]}\;\phi_{0}^{\;\left[3\right]}\;\phi_{0}^{\;\left[4\right]}\right]$. Under the dynamic multistate occupancy model, state probabilities for subsequent seasons *t* (*t* = 1,.., *T*) depend on the true state in season *t*-1. As such, occupancy dynamics are incorporated in the model by considering transition probabilities from one true state to another across years. We denote the probability of transitioning from state *m* at time *t* to state *n* at time *t*+1 with $\phi_{t}^{\left[m,n\right]}$. A transition probability matrix (TPM) defines the probability of each true state at time *t*+1 given the possible true states at time *t*. For example, a four-state transition probability matrix may be represented as follows:

$$\Phi_{t}=\left[\begin{array}{cccc} \phi_{t}^{\left[1,1\right]} & \phi_{t}^{\left[1,2\right]} & \phi_{t}^{\left[1,3\right]} & \phi_{t}^{\left[1,4\right]}\\ \phi_{t}^{\left[2,1\right]} & \phi_{t}^{\left[2,2\right]} & \phi_{t}^{\left[2,3\right]} & \phi_{t}^{\left[2,4\right]}\\ \phi_{t}^{\left[3,1\right]} & \phi_{t}^{\left[3,2\right]} & \phi_{t}^{\left[3,3\right]} & \phi_{t}^{\left[3,4\right]}\\ \phi_{t}^{\left[4,1\right]} & \phi_{t}^{\left[4,2\right]} & \phi_{t}^{\left[4,3\right]} & \phi_{t}^{\left[4,4\right]} \end{array}\right]$$

Each row in the TPM sums to 1, and represents the state probability vector at time *t*+1 given the row state at time *t*; i.e., the transition probabilities from time *t* to *t*+1 conditional on the state at time *t*. In all cases the true state of a territory is assumed to remain constant within a season.

We denote the probability of observing a territory in state *n* during survey *j* of year *t*, given a true occupancy state *m* as $p_{t,j}^{m,n}$. Uncertainty in observed states is assumed to be constrained such that $p_{t,j}^{m,n}=0$ for any *n* > *m*. For example, if the true state were 2, we assume that it is not possible to detect the species in either state 3 or 4, but that either state 1 or 2 could be observed. A four state detection probability matrix (DPM) may then be defined as:

$$\mathrm{True State}\;\bar{|\begin{array}{ccccc} \; & 1 & 2 & 3 & 4\\ 1 & 1 & 0 & 0 & 0\\ 2 & 1-p_{tj}^{2,2} & p_{tj}^{2,2} & 0 & 0\\ 3 & 1-p_{tj}^{3,2}-p_{tj}^{3,3} & p_{tj}^{3,2} & p_{tj}^{3,3} & 0\\ 4 & 1-p_{tj}^{4,2}-p_{tj}^{4,3}-p_{tj}^{4,4} & p_{tj}^{4,2} & p_{tj}^{4,3} & p_{tj}^{4,4} \end{array}}$$

Under the MacKenzie et al. [23] model, the observed state of a territory is taken to be a random vector **y** which, conditional on the true state **z**, is assumed to arise as a single draw from a multinomial distribution. For example, if the true state of territory *i* in year *t* was a non-reproducing pair (state 3), then the multinomial probability vector for the observed state at visit *j* would be $\left[1-p_{tj}^{3,2}-p_{tj}^{3,3}\;p_{tj}^{3,2}\;p_{tj}^{3,3}\;0\right]$, so that the probability of observing a single owl in this case would be $p_{tj}^{3,2}$.

In our analysis of the spotted owl data, we did not include any covariates in the TPM or the initial state probabilities, as none were available and our goal was to estimate the number of territories and long-term trends. For the initial state probabilities, we specified a Dirichlet prior distribution with all parameters equal to 1, equivalent to a multivariate uniform distribution. Using random effects, we allowed for the values of $\phi_{t}^{\left[m,n\right]}$ to vary by season, yet also allow for similarity across seasons. For example, $\phi_{t}^{\left[m,n\right]}$ may tend to be low across most seasons for some values of *m* and *n*, while for other values of *m* and *n* it may tend to be moderate across most seasons. Similarly, some parameters $\phi_{t}^{\left[m,n\right]}$ may tend to show little variation over time, while others may tend to show a larger degree of variation over time. We incorporate this added structure via random effects using the multi-logit transformation (to ensure row probabilities sum to 1). Specifically, we assumed

$$\phi_{t}^{\left[m,n\right]}=\text{Pr}\left(z_{t}=n{|\text{z}}_{t-1}=m\right)=\frac{\text{exp}\left(\beta_{0,t}^{\left[m,n\right]}\right)}{1+\Sigma_{l=2}^{4}\;\text{exp}\left(\beta_{0,t}^{\left[m,l\right]}\right)}\;\text{for}\;n>1,\;\text{and}$$

$$\phi_{t}^{\left[m,n\right]}=\text{Pr}\left(z_{t}=1{|\text{z}}_{t-1}=m\right)=\frac{1}{1+\Sigma_{l=2}^{4}\;\text{exp}\left(\beta_{0,t}^{\left[m,l\right]}\right)}\;,$$

where the random effects (intercept only) were assumed to arise from a normal distribution:

$$\beta_{0,t}^{\left[m,n\right]}∼N\left(\mu^{\left[m,n\right]},\sigma^{\left[m,n\right]}\right)\;\text{for}\;n>1.$$

The random effects mean and variance, *μ*^[*m*,*n*]^ and *σ*^[*m*,*n*]^, were specified with N(0, 2) and Gamma(2, 0.5) prior distributions, respectively, for all values of *m* and *n*. We chose these priors to provide broad prior distributions for the transition probability parameters $\phi_{t}^{\left[m,n\right]}$. The use of random effects provides improved precision of annual estimates of the TPM.

The dates on which owl surveys were conducted varied across owl territories and across years. To allow for within-season variability in detection probabilities, we parameterized each (non-constant) element of the DPM with linear and quadratic Julian date terms. Further, we allowed the date effects to vary across seasons by incorporating random effects for the coefficients. Specifically, we assumed for *m* > 1 that:

$$p_{itj}^{\left[m,n\right]}=\text{Pr}\left(y_{itj}=n|z_{it}=m\right)=\frac{\text{exp}\left(\alpha_{0,t}^{\left[m,n\right]}+\alpha_{1,t}^{\left[m,n\right]}\cdot {\text{JD}}_{ij}+\alpha_{2,t}^{\left[m,n\right]}\cdot {\text{JD}}_{{}_{ij}}^{2}\right)}{1+\Sigma_{l=2}^{m}\text{exp}\left(\alpha_{0,t}^{\left[m,l\right]}+\alpha_{1,t}^{\left[m,l\right]}\cdot {\text{JD}}_{ij}+\alpha_{2,t}^{\left[m,l\right]}\cdot {\text{JD}}_{{}_{ij}}^{2}\right)}\;\text{for}\;1<n\leq m,\;\text{and}$$

$$p_{itj}^{\left[m,1\right]}=\text{Pr}\left(y_{itj}=1|z_{it}=m\right)=\frac{1}{1+\Sigma_{l=2}^{m}\;\text{exp}\left(\alpha_{0,t}^{\left[m,l\right]}+\alpha_{1,t}^{\left[m,l\right]}\cdot {\text{JD}}_{ijt}+\alpha_{2,t}^{\left[m,l\right]}\cdot {\text{JD}}_{{}_{ijt}}^{2}\right)},$$

with normal random effects for the intercept, linear and quadratic terms:

$$\alpha_{0,t}^{\left[m,n\right]}∼N\left(\mu_{0}^{\left[m,n\right]},\sigma_{0}^{2\left[m,n\right]}\right)$$

$$\alpha_{1,t}^{\left[m,n\right]}∼N\left(\mu_{1}^{\left[m,n\right]},\sigma_{1}^{2\left[m,n\right]}\right)$$

$$\alpha_{2,t}^{\left[m,n\right]}∼N\left(\mu_{2}^{\left[m,n\right]},\sigma_{2}^{2\left[m,n\right]}\right).$$

Here, *y*_*itj*_ is the observed state for visit *j*, year *t*, territory *i*; *z*_*it*_ is the true state of territory *i* in year *t*; and JD_*itj*_ is the Julian date for territory *i* during visit *j* in year *t*. We specified prior distributions for the DPM random effects mean and variance as N(0,2) and Gamma(2, 0.5), respectively. We centered and scaled Julian data prior to analysis.

We collected data for this analysis on territories distributed across three management blocks, each with its own management history (D. Meekins, pers. comm.). We allowed all parameters of our model to vary by block in order to obtain block-specific estimates of quantities of interest.

Although the dynamic multistate occupancy model is parameterized in terms of an initial state probability vector and transition probabilities, estimates of state probabilities may be obtained for any given season through the recursive matrix operation:

$$\phi_{t}=\phi_{t-1}\phi_{t-1}$$

An advantage of the state-space approach from MacKenzie et al. [23] is that the true state of a territory is imputed for each season, allowing for summaries of territory states for a given territory or across the population sample. For example, one can obtain posterior mean estimates of the number of reproductive territories during each season *t*, along with posterior credible intervals. In turn, the seasonal summaries allow for the estimation of long term linear or quadratic trends across seasons using, for example, orthogonal polynomial contrasts [24]. In our study, we calculated linear trends for number of reproductive territories using the estimator:

$$\tilde{T_{L}}=\frac{1}{S}\Sigma_{s=1}^{S}T_{L}^{\left(s\right)},\;\text{where}\;T_{L}^{\left(s\right)}=\frac{\Sigma_{t}N_{{}_{t}}^{\left(s\right)}\left(Yr_{t}-\bar{Yr}\right)}{\Sigma_{t}{\left(Yr_{t}-\bar{Yr}\right)}^{2}}.$$

To calculate ${\tilde{T}}_{L,}$ a set of s = 1, …, S simulations were sampled from the posterior distribution. The quantity $N_{{}_{t}}^{\left(s\right)}$ is the *s*-th sample of the total number of reproductive territories imputed at time *t* (does not include pair or single territories), *Yr*_*t*_ is the year at time t, and $\bar{Yr}$ is the mean year. $T_{L}^{\left(s\right)}$ is calculated from the *s*-th sample of the posterior distribution, and we take the average over all samples to calculate an estimate of the posterior mean. Credible intervals may be calculated in a similar manner using sample quantiles for $T_{L}^{\left(s\right)}$. The estimator ${\tilde{T}}_{L,}$ is interpreted as the expected average change in the number of reproductive territories over each one year interval. For example, a value of -1.0 would indicate an average decline of 1 reproductive territory per year over the course of the study.

We fit the model using JAGS [25] called from R [26] using package R2jags [27]. We ran three chains of length 55,000 with a burnin of 5,000 and 1/100 thinning. Convergence was assessed using the Gelman-Rubin statistic [28] and visual inspection of the chains, with results consistent with Markov chain convergence. We used posterior predictive checks to assess agreement between the fitted model and the observed data, and these checks did not indicate problems with the fitted model (steps in S1 File; results in S1 Table). R code for specifying the JAGS model is included in S2 File. Data used in analyses are in S2 Table.

### Ethics Statement

We conducted this research in compliance with all California and USA laws and regulations. The United States Fish and Wildlife Service (USFWS) approved all activities involving the sampling and handling of live vertebrate animals.

## Results

**Fig 1:**
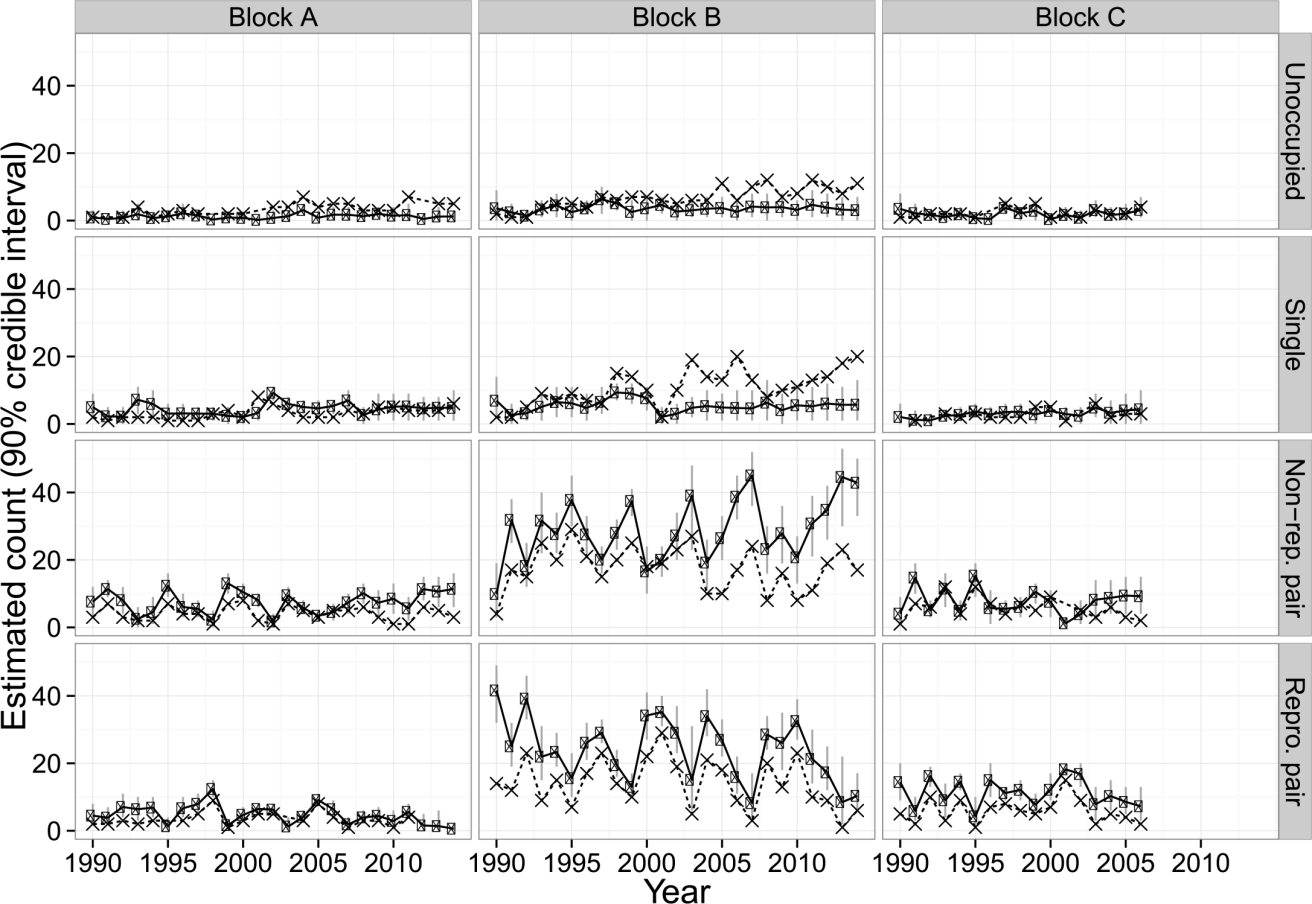
Estimated number of northern spotted owl territories (‘•’ symbol with 90% CRI) in each of four occupancy states by management block and year, Mendocino County, CA, USA, 1990–2014. Estimates shown with the ‘×’ symbol are the naïve counts (i.e., unadjusted for imperfect detection) of territories in each occupancy state, and do not show credible intervals.

We found substantial annual variation in the number of territories in States 3 and 4 while the number of territories in States 1 and 2 remained relatively constant across years (Fig 1). We note that use of naïve territory counts would have over-estimated the number of territories in State 2 in Block B (Fig 1). As a result, the number of territories occupied by pairs in a given year would have been underestimated. Estimated (linear) trends in the number of reproductive territories suggest an average decline of approximately 0.15 territories every year in Block A, and one half territory per year in Block B over the 25 years of this study (1990–2014; Table 1). The estimated per-year trend (-0.09) for Block C was also negative (Table 1). However, for Block C, 90% credible intervals contained zero, indicating uncertainty as to the direction of this trend. Trends in the combined number of pair and reproductive pair territories showed no clear evidence of an increase or decrease in any of the three study blocks. Estimates from 2002–2014 indicated that both Block A and Block B may have lost reproductive pairs, with an estimated decline of approximately 0.32 and 0.97 nesting pairs per year, respectively. However, in both cases the number of pair and reproductive pairs combined showed no clear trends during the same period (Table 1). We note that the size of the estimated decline in number of reproductive pairs was greater for Blocks A and B from 2002–2014 than from 1990–2014, suggesting that the magnitude of the decline increased in the second half of the study period.

**Table 1:** Trend estimates and 90% posterior credible intervals for the number of northern spotted owl territories occupied by reproductive pairs and pairs and reproductive pairs combined on three management blocks, Mendocino County, CA, USA, 1990–2014. We estimated contrasts from 1990–2002, 2002–2014, and 1990–2014. Block C was not surveyed after 2006. As a result, we estimated trends for Block C for 1990–2002 and 1990–2006 only. Mean estimates represent the expected change in territory count for a one year interval.

Management block	Years	State	Trend estimates (90% posterior interval)
A	1990–2002	Reproductive pairs only	0.10 (-0.12, 0.28)
B	1990–2002	Reproductive pairs only	-0.35 (-0.87, 0.17)
C	1990–2002	Reproductive pairs only	0.31 (0.01, 0.62)
A	1990–2002	Pairs and reproductive pairs	-0.02 (-0.23, 0.21)
B	1990–2002	Pairs and reproductive pairs	-0.15 (-0.48, 0.21)
C	1990–2002	Pairs and reproductive pairs	-0.06 (-0.30, 0.18)
A	2002–2014	Reproductive pairs only	-0.32 (-0.49, -0.14)
B	2002–2014	Reproductive pairs only	-0.97 (-1.6, -0.40)
C	2002–2006	Reproductive pairs only	NA
A	2002–2014	Pairs and reproductive pairs	0.24 (-0.04, 0.53)
B	2002–2014	Pairs and reproductive pairs	-0.19 (-0.61, 0.20)
C	2002–2006	Pairs and reproductive pairs	NA
A	1990–2014	Reproductive pairs only	-0.15 (-0.24, -0.07)
B	1990–2014	Reproductive pairs only	-0.55 (-0.76, -0.33)
C	1990–2006	Reproductive pairs only	-0.09 (-0.35, 0.17)
A	1990–2014	Pairs and reproductive pairs	-0.07 (-0.20, 0.05)
B	1990–2014	Pairs and reproductive pairs	-0.01 (-0.19, 0.16)
C	1990–2006	Pairs and reproductive pairs	-0.16 (-0.40, 0.06)

**Fig 2:**
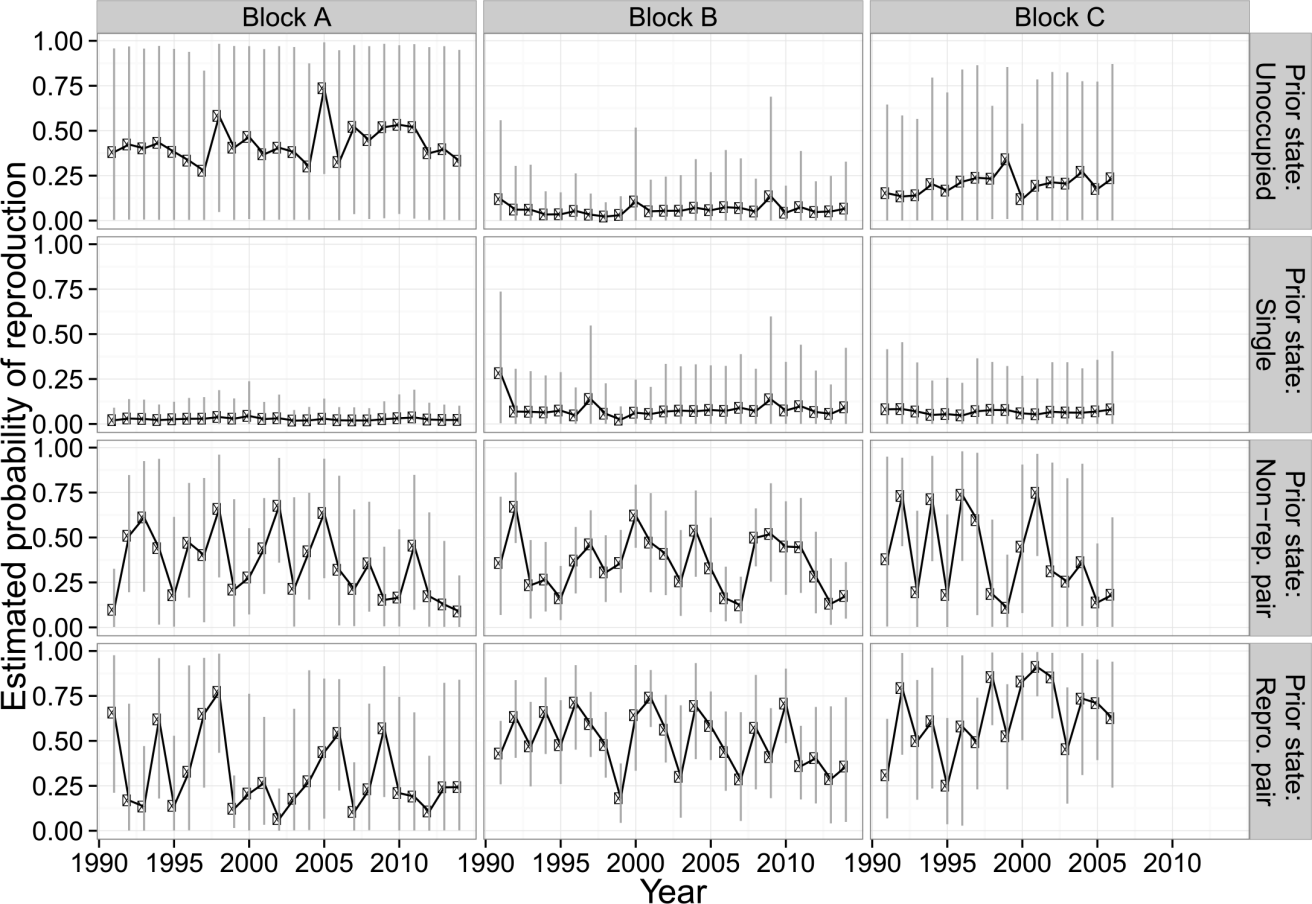
Estimated probability (90% CRI) of reproduction in each management block and year given the occupancy state of a territory in the previous year for northern spotted owls, Mendocino County, CA, USA, 1990–2014.

In all three management blocks, we found substantial variation in probability of reproduction, depending on prior territory state (Fig 2). Averaged across all years of the study (using hyper-prior means), probability of reproducing in a current year given reproduction in a previous year was 0.30 (90% CRI: 0.13, 0.49), 0.50 (90% CRI: 0.40, 0.60), and 0.70 (90% CRI: 0.53, 0.85) across Blocks A, B, and C, respectively. In contrast, probability of reproducing in a current year, given non-reproduction in a previous year, was 0.30 (90% CRI: 0.16, 0.46), 0.34 (90% CRI: 0.25, 0.43), and 0.37 (90% CRI: 0.14, 0.60) across Blocks A, B, and C, respectively. Estimates for all blocks indicated that probability of reproduction (State 4) was rare if a territory was occupied by a single owl (State 2) in the previous year (Fig 2). Estimates for Block B indicated that probability of reproduction (State 4) was rare if a territory was unoccupied (State 1) in the previous year. Data were insufficient to estimate reproduction when the state in the previous year was unoccupied (State 1) for Blocks A and Block C.

Additionally, we found considerable variation in reproductive probability across years for owl pairs (Fig 2). For example, mean estimates of reproductive probability, with prior-year reproduction, in Block A ranged from a high of 0.76 to a low of 0.06, in Block B from 0.20 to 0.74, and in Block C from 0.25 to 0.92. Similar variability in reproductive probability was seen when the prior state was a non-reproductive pair. Although years with low reproductive probability were often followed by years with higher probability, this was not always the case; some 4–5 year spans have increasing estimates of reproduction, and other 4–5 year spans have decreasing estimates of reproduction (Fig 2).

**Fig 3:**
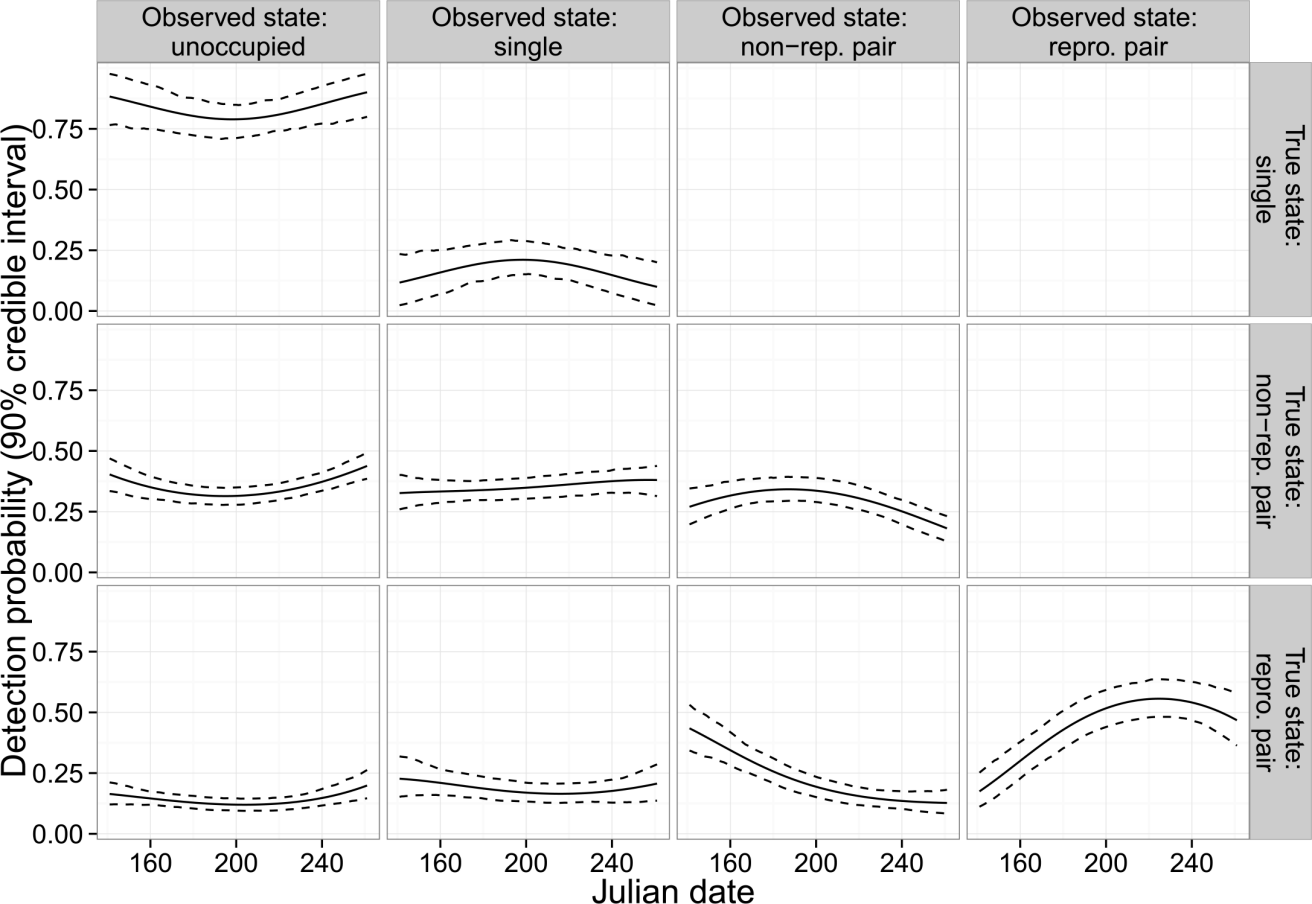
Estimated survey-specific detection probabilities (90% CRI) as a function of Julian date for northern spotted owls, Mendocino County, CA, USA, 1990–2014. We calculated estimates from a hierarchical model in which the quadratic parameters for Julian date varied by year using a random effects specification. The trends displayed in this graphic use the mean posterior random effects.

Estimated probabilities of observing the true state were highest for reproductive pairs (State 4) and lowest for single owls (State 2; Fig 3). Similarly, the estimated probability of observing no owls was highest for single owls and lowest for reproductive pairs. The seasonal trend in detection probability was most pronounced for reproductive pairs, with the highest probability of detection occurring in the first week of August across all years. Survey-specific detection probabilities for State 2 were low (≤ 0.25 for all years). However, all surveys included in this analysis occurred during the day (in order to assess reproductive status). Typical annual surveys of NSO territories include a mix of day and night surveys and will likely be more effective at detecting both single and pairs of owls.

## Discussion

Long-term datasets provide critical information about management and conservation options for imperiled species, including those affected by habitat loss and/or competition with invasive species. We did not find evidence to indicate that territory occupancy by northern spotted owl pairs declined substantially over a 25 year period on the Mendocino County, CA, study area. These results stand in marked contrast to other studies that also examined northern spotted owl occupancy dynamics over extended time frames and found evidence indicating significant declines [9, 11–15]. Although we estimated a declining trend in territory occupancy by breeding pairs, particularly over the last 12 years, we note that due to cyclical reproductive patterns long-term trends are influenced by the interval over which trends are estimated. For example, had monitoring began in either 1994 or 1995, and ended in 2010 or 2011, we would have concluded that territory occupancy by breeding pairs had remained constant or possibly increased. In addition, we note that similarly low numbers of reproductive pairs were observed in 1999, 2003, and 2007. Finally, consistent with most long-term datasets for northern spotted owls, the initial sample consisted of territories that were either occupied or had been occupied recently [11, 15], rather than a random sample from a ‘population’ of unoccupied and occupied northern spotted owl territories. As a result, our initial estimate of territory occupancy by breeding pairs may have been inflated compared to the background occupancy rate for this particular state.

Our study differs markedly from other long-term investigations of NSO populations in three ways: forest type, management prescriptions, and absence of barred owls. First, all three study blocks occur in the coastal Redwood forest zone; other long-term occupancy studies have occurred in mixed conifer or western hemlock zones [29]. This forest type has different structural characteristics than other single-species dominated or mixed-conifer stands within the range of the northern spotted owl, including understory plant diversity and structure and potential to develop and retain large amounts of coarse woody debris [17, 18]. Second, stands on the three blocks have been managed intensively for timber production for over 100 years. As a result, the study area is dominated by stands less than 80 years in age. Other long-term NSO study areas are composed primarily of Federal lands managed for natural values [11, 12] and contain substantial amounts of late-successional forest, although some exceptions do exist [14]. Finally, we detected only 25 barred owls in 25 years of intensive surveys using the same methods that detect barred owls regularly throughout the range of the northern spotted owl. Given that 15 (60%) of these detections occurred from 1990–1999, when the range of the barred owl had not yet enveloped that of the northern spotted owl, we conclude that barred owls have had ample opportunity to colonize the three management blocks. We cannot determine why barred owls have not colonized our study area, although forest type, management history and contemporary practices, and climatic conditions‒and interactions between all three factors‒may influence this result. Similarly, we cannot preclude the possibility that barred owls will colonize some or the majority of the northern spotted owl territories in our study area in the future (as barred owls have done in other study areas).

Similar to other studies, we found a pronounced pattern in the annual reproductive activity of northern spotted owls [10, 23, 30]. In this case, the pattern was manifested as the regular transition of territories between States 3 and 4. Other studies have attributed this pattern to climatic variation early in the annual nesting cycle, usually elevated amounts of precipitation [30–32]. Given its immediate proximity to the Pacific Ocean, our study area can experience extremely variable spring precipitation [33]. Our data provide mixed support for the association between reproduction and local climatic variation. For example, occupancy of reproductive pairs was the lowest in 2003, 2007, 2013, and 2014; March precipitation in those four years was 14.9, 3.9, 8.1, and 21.5 cm, respectively, equating to 111, 29, 61, and 161% of the long-term average [34]. Annual variation in northern spotted owl reproductive effort has also been attributed to variation in prey abundance [35] and female age [30]. We did not include this covariate information in our modeling effort, but recognize that this information, as well as climatic variables, may have strong associations with occupancy states and recommend their inclusion in future analyses.

Occupancy and multi-season occupancy models [36, 37] that account for imperfect detection have become standard methods for the analysis of spotted owl data [11, 13, 15, 38]. We think that multistate occupancy modeling [23, 39] offers several advantages over other methods, and warrants serious consideration by researchers conducting spotted owl research when suitable data are available for analysis. For example, multistate modeling provides a more detailed picture of population dynamics, allowing for inference about processes such as reproductive dynamics. The state-space approach for multistate modeling imputes the number of territories for each nesting state, which in a longitudinal study allows for the estimation of longer-term trends in occupied or reproductive territories. A particular benefit of the multistate approach is the allowance for different detection probabilities by nesting state‒to account for either inherent differences in detection or for the use of different survey methods for different occupancy states‒as was done in this study. For example, our estimated probabilities for observing an unoccupied territory varied substantially across each of the three occupied states (Fig 3). Further, the estimated probability of observing a single-owl territory was higher for non-reproductive territories than for reproductive territories in our study. Such differences may lead to bias in studies for which multiple states are collapsed to a single level, e.g., so-called “simple” analyses [11, 15].

Although we found many benefits to the multistate approach, these models present substantial challenges. Our parameterization was relatively simple, in keeping with the goals of the analysis and the management history of the study territories. However, a researcher interested in the potential effects of multiple covariates may face difficult choices regarding how to parameterize the model. For example, in an analysis with M states, M*(M-1) free elements exist in the TPM and M*(M-1)/2 free elements exist in the DPM. Each of these elements could include covariates, and the researcher must determine which covariates to include in each element as well as the functional form they would take. Another challenge relates to assessing adequacy-of-fit. We chose our approach (S1 File) to evaluate how well our model predicted observed patterns in *detected* states. Ultimately, we cannot disentangle process variation and detection probability with absolute confidence, meaning that such an approach may leave uncovered important problems with the model. Further development of adequacy-of-fit approaches and tests of closure for dynamic multistate occupancy models would help researchers undertake the important step of model checking.

Our results indicate that the number of territories occupied by northern spotted owl pairs remained relatively constant over a 25 year period in a landscape with an extensive legacy of historic, as well as contemporary, timber management. However, we cannot exclude small-to-moderate declines or increases in paired territory numbers due to uncertainty in our estimates. Given the stated close association of northern spotted owls with late-successional forest in other parts of their range [10, 40, 41], these results suggest that habitat per se may not be the only factor that determines population performance of this endangered raptor. For instance, a recent meta-analysis [9] found strong declines in northern spotted owl occupancy on study areas that contained substantial amounts of older forest as well as large populations of barred owls. Taken together, these results indicate that habitat conditions explain only some of the variation in northern spotted owl occupancy. Finally, given proposed removals of barred owls in other portions of the range of the northern spotted owl [9, 16], the Mendocino County population evaluated in this study serves as a control population. As such, it provides long-term insight into northern spotted owl occupancy states in the absence of an exotic congener known to exert significant negative effects, the barred owl.

## Supporting Information

S1 Fig Location of management blocks A, B, and C, northern spotted owl study area, Mendocino County, CA, USA, 1990–2014.
(EPS)

S1 File Steps used to perform posterior predictive checks for model diagnostics. (DOCX)

S2 File R code for JAGS specification of the dynamic multistate occupancy model. (DOCX)

S1 Table Output from model diagnostics from simulation studies for dynamic multistate occupancy model. (XLSX)

S2 Table Data used in dynamic multistate occupancy model for northern spotted owls, Mendocino County, CA, USA, 1990–2014. (XLSX)
