# Supplementary material for: Multistate Models Reveal Long-Term Trends of Northern Spotted Owls in the Absence of a Novel Competitor
Source: PLoS One. 2016 Apr 11;11(4):e0152888. doi: 10.1371/journal.pone.0152888 (PMC4827817; doi:10.1371/journal.pone.0152888)
Supplement: S1 File — (DOCX) [file pone.0152888.s002.docx]

**S1 File.** Steps used to perform posterior predictive checks for model diagnostics. We used posterior predictive checks (Gelman et al., 2004) to compare how closely data simulated from the fitted model agree with the observed data. We compare the proportion of detected (or ‘naïve’) states across years in the observed data with the distribution of naïve states simulated from the model. By ‘naïve’, we mean the maximum state index over all visits to a given site within a season (i.e., the state we would use if not accounting for imperfect detection).

Let:

$y_{ijk}=$ observed state at site *i*, year *j*, visit *k*

$y_{ij.}=\max_{k}\left(y_{ijk}\right)=$ ‘naïve’ state index at site *i*, year *j*

$y_{ijk}^{\left(s\right)}=$ the *s*^th^ simulated observed state at site *i*, year *j*, visit *k*

$y_{ij.}^{\left(s\right)}=\text{max}_{k}y_{ijk}^{\left(s\right)}=$ the *s*^th^ simulated ‘naïve’ state index at site *i*, year *j*.

Simulate $y_{ijk}^{\left(s\right)}$ as follows:

1. Draw an imputed state vector $z_{ij}^{\left(s\right)}$from the posterior distribution. This is the simulated ‘true’ state.
2. Draw detection parameters$\theta_{ijk}^{\left(s\right)}$from the posterior distribution.
3. Construct the detection probability matrix using $\theta_{ijk}^{\left(s\right)}$
4. Extract the detection probability vector appropriate to $z_{ij}^{\left(s\right)}$from the detection probability matrix.
5. Draw a random value of $y_{ijk}^{\left(s\right)}$from a multinomial distribution with size = 1 and probability vector from Step 4.
6. Repeat *S* times.

Diagnostic checks are performed by calculating the observed proportion of each state at each time period j:

$\pi_{m,j}=\frac{\sum_{i} I\left(y_{ij.}=m\right)}{N},$ where *N*=number of territories, and *m*=state of interest, and comparing this quantity to the distribution of state proportions simulated from the model:

$$\pi_{m,j}^{\left(s\right)}=\frac{\sum_{i} I\left(y_{ijk}^{\left(s\right)}=m\right)}{N}.$$

Output from these checks are presented in Table S3.
