# Supplementary material for: Multistate Models Reveal Long-Term Trends of Northern Spotted Owls in the Absence of a Novel Competitor
Source: PLoS One. 2016 Apr 11;11(4):e0152888. doi: 10.1371/journal.pone.0152888 (PMC4827817; doi:10.1371/journal.pone.0152888)
Supplement: S2 File — (DOCX) [file pone.0152888.s003.docx]

**S2 File**: R code for JAGS specification of the dynamic multistate occupancy model for northern spotted owls, Mendocino County, CA, USA, 1990-2014. For additional details, see MacKenzie, D.I., J.D. Nichols, M.E. Seamans, and R.J. Gutiérrez. 2009. Modeling species occurrence dynamics with multiple states and imperfect detection. *Ecology* 90:823-835.

# TPM and DPM use a multi-logit parameterization (Hastie et al. 2009, Ch. 4) to ensure that row probabilities sum to 1.

# Hastie, T., R. Tibshirani, and J. Friedman. 2009. The Elements of Statistical Learning, 2^nd^ edition. Springer, New York, NY, USA.

#

model <- function(){

# model with 4 states that vary by block x year, and detection

# probabilities that vary by year and julian date. Random

# effects used to stabilize parameter estimates across years.

# Note: 1=Unocc, 2=Single, 3=Pair (non-repro), 4=Pair

#

### Transition probability matrix (TPM) parameters

# allow TPM parameters to vary by batch and by year

for(bb in 1:nBlk){

## initial state

Phi[bb,1:4] ~ ddirch(alpha4[])

## hyper-priors for TPM parameters

# from state=unocc

phi12mean[bb] ~ dnorm(0, 0.333)

phi13mean[bb] ~ dnorm(0, 0.333)

phi14mean[bb] ~ dnorm(0, 0.333)

phi12var[bb] ~ dgamma(2, 0.5)

phi13var[bb] ~ dgamma(2, 0.5)

phi14var[bb] ~ dgamma(2, 0.5)

phi12tau[bb] <- 1/phi12var[bb]

phi13tau[bb] <- 1/phi13var[bb]

phi14tau[bb] <- 1/phi14var[bb]

# from state=single

phi22mean[bb] ~ dnorm(0, 0.333)

phi23mean[bb] ~ dnorm(0, 0.333)

phi24mean[bb] ~ dnorm(0, 0.333)

phi22var[bb] ~ dgamma(2, 0.5)

phi23var[bb] ~ dgamma(2, 0.5)

phi24var[bb] ~ dgamma(2, 0.5)

phi22tau[bb] <- 1/phi22var[bb]

phi23tau[bb] <- 1/phi23var[bb]

phi24tau[bb] <- 1/phi24var[bb]

# from state=pair

phi32mean[bb] ~ dnorm(0, 0.333)

phi33mean[bb] ~ dnorm(0, 0.333)

phi34mean[bb] ~ dnorm(0, 0.333)

phi32var[bb] ~ dgamma(2, 0.5)

phi33var[bb] ~ dgamma(2, 0.5)

phi34var[bb] ~ dgamma(2, 0.5)

phi32tau[bb] <- 1/phi32var[bb]

phi33tau[bb] <- 1/phi33var[bb]

phi34tau[bb] <- 1/phi34var[bb]

# from state=repro

phi42mean[bb] ~ dnorm(0, 0.333)

phi43mean[bb] ~ dnorm(0, 0.333)

phi44mean[bb] ~ dnorm(0, 0.333)

phi42var[bb] ~ dgamma(2, 0.5)

phi43var[bb] ~ dgamma(2, 0.5)

phi44var[bb] ~ dgamma(2, 0.5)

phi42tau[bb] <- 1/phi42var[bb]

phi43tau[bb] <- 1/phi43var[bb]

phi44tau[bb] <- 1/phi44var[bb]

# allow TPM parameters to vary by year

for(tt in 1:T-1){

# from state=unocc

logPhi12[bb,tt] ~ dnorm(phi12mean[bb], phi12tau[bb])

logPhi13[bb,tt] ~ dnorm(phi13mean[bb], phi13tau[bb])

logPhi14[bb,tt] ~ dnorm(phi14mean[bb], phi14tau[bb])

# from state=single

logPhi22[bb,tt] ~ dnorm(phi22mean[bb], phi22tau[bb])

logPhi23[bb,tt] ~ dnorm(phi23mean[bb], phi23tau[bb])

logPhi24[bb,tt] ~ dnorm(phi24mean[bb], phi24tau[bb])

# from state=pair

logPhi32[bb,tt] ~ dnorm(phi32mean[bb], phi32tau[bb])

logPhi33[bb,tt] ~ dnorm(phi33mean[bb], phi33tau[bb])

logPhi34[bb,tt] ~ dnorm(phi34mean[bb], phi34tau[bb])

# from state=repro

logPhi42[bb,tt] ~ dnorm(phi42mean[bb], phi42tau[bb])

logPhi43[bb,tt] ~ dnorm(phi43mean[bb], phi43tau[bb])

logPhi44[bb,tt] ~ dnorm(phi44mean[bb], phi44tau[bb])

}

# populate the TPM

for(tt in 1:T-1){

TPM[bb,tt,1,1] <- 1/(1+exp(logPhi12[bb,tt])+exp(logPhi13[bb,tt])+exp(logPhi14[bb,tt])) # from Unocc to Unocc

TPM[bb,tt,1,2] <- exp(logPhi12[bb,tt])/(1+exp(logPhi12[bb,tt])+exp(logPhi13[bb,tt])+exp(logPhi14[bb,tt])) # from Unocc to Single

TPM[bb,tt,1,3] <- exp(logPhi13[bb,tt])/(1+exp(logPhi12[bb,tt])+exp(logPhi13[bb,tt])+exp(logPhi14[bb,tt])) # from Unocc to Pair (non-repro)

TPM[bb,tt,1,4] <- exp(logPhi14[bb,tt])/(1+exp(logPhi12[bb,tt])+exp(logPhi13[bb,tt])+exp(logPhi14[bb,tt])) # from Unocc to Repro

TPM[bb,tt,2,1] <- 1/(1+exp(logPhi22[bb,tt])+exp(logPhi23[bb,tt])+exp(logPhi24[bb,tt])) # from Single to Unocc

TPM[bb,tt,2,2] <- exp(logPhi22[bb,tt])/(1+exp(logPhi22[bb,tt])+exp(logPhi23[bb,tt])+exp(logPhi24[bb,tt])) # from Single to Single

TPM[bb,tt,2,3] <- exp(logPhi23[bb,tt])/(1+exp(logPhi22[bb,tt])+exp(logPhi23[bb,tt])+exp(logPhi24[bb,tt])) # from Single to Pair

TPM[bb,tt,2,4] <- exp(logPhi24[bb,tt])/(1+exp(logPhi22[bb,tt])+exp(logPhi23[bb,tt])+exp(logPhi24[bb,tt])) # from Single to Repro

TPM[bb,tt,3,1] <- 1/(1+exp(logPhi32[bb,tt])+exp(logPhi33[bb,tt])+exp(logPhi34[bb,tt])) # from Pair (non-repro) to Unocc

TPM[bb,tt,3,2] <- exp(logPhi32[bb,tt])/(1+exp(logPhi32[bb,tt])+exp(logPhi33[bb,tt])+exp(logPhi34[bb,tt])) # from Pair to Single

TPM[bb,tt,3,3] <- exp(logPhi33[bb,tt])/(1+exp(logPhi32[bb,tt])+exp(logPhi33[bb,tt])+exp(logPhi34[bb,tt])) # from Pair to Pair

TPM[bb,tt,3,4] <- exp(logPhi34[bb,tt])/(1+exp(logPhi32[bb,tt])+exp(logPhi33[bb,tt])+exp(logPhi34[bb,tt])) # from Pair to Repro

TPM[bb,tt,4,1] <- 1/(1+exp(logPhi42[bb,tt])+exp(logPhi43[bb,tt])+exp(logPhi44[bb,tt])) # from Repro to Unocc

TPM[bb,tt,4,2] <- exp(logPhi42[bb,tt])/(1+exp(logPhi42[bb,tt])+exp(logPhi43[bb,tt])+exp(logPhi44[bb,tt])) # from Repro to Single

TPM[bb,tt,4,3] <- exp(logPhi43[bb,tt])/(1+exp(logPhi42[bb,tt])+exp(logPhi43[bb,tt])+exp(logPhi44[bb,tt])) # from Repro to Pair

TPM[bb,tt,4,4] <- exp(logPhi44[bb,tt])/(1+exp(logPhi42[bb,tt])+exp(logPhi43[bb,tt])+exp(logPhi44[bb,tt])) # from Repro to Repro

}

}

### Detection probability matrix parameters

# hyper-priors if state=single

mu220 ~ dnorm(mu, tau) # intercept

mu221 ~ dnorm(mu, tau) # linear JD

mu222 ~ dnorm(mu, tau) # quadratic JD

var220 ~ dgamma(2, 0.5)

var221 ~ dgamma(2, 0.5)

var222 ~ dgamma(2, 0.5)

tau220 <- 1/var220

tau221 <- 1/var221

tau222 <- 1/var222

# hyper-priors if state=pair

mu320 ~ dnorm(mu, tau)

mu321 ~ dnorm(mu, tau)

mu322 ~ dnorm(mu, tau)

var320 ~ dgamma(2, 0.5)

var321 ~ dgamma(2, 0.5)

var322 ~ dgamma(2, 0.5)

tau320 <- 1/var320

tau321 <- 1/var321

tau322 <- 1/var322

mu330 ~ dnorm(mu, tau)

mu331 ~ dnorm(mu, tau)

mu332 ~ dnorm(mu, tau)

var330 ~ dgamma(2, 0.5)

var331 ~ dgamma(2, 0.5)

var332 ~ dgamma(2, 0.5)

tau330 <- 1/var330

tau331 <- 1/var331

tau332 <- 1/var332

# hyper-priors for state=repro

mu420 ~ dnorm(mu, tau)

mu421 ~ dnorm(mu, tau)

mu422 ~ dnorm(mu, tau)

var420 ~ dgamma(2, 0.5)

var421 ~ dgamma(2, 0.5)

var422 ~ dgamma(2, 0.5)

tau420 <- 1/var420

tau421 <- 1/var421

tau422 <- 1/var422

mu430 ~ dnorm(mu, tau)

mu431 ~ dnorm(mu, tau)

mu432 ~ dnorm(mu, tau)

var430 ~ dgamma(2, 0.5)

var431 ~ dgamma(2, 0.5)

var432 ~ dgamma(2, 0.5)

tau430 <- 1/var430

tau431 <- 1/var431

tau432 <- 1/var432

mu440 ~ dnorm(mu, tau)

mu441 ~ dnorm(mu, tau)

mu442 ~ dnorm(mu, tau)

var440 ~ dgamma(2, 0.5)

var441 ~ dgamma(2, 0.5)

var442 ~ dgamma(2, 0.5)

tau440 <- 1/var440

tau441 <- 1/var441

tau442 <- 1/var442

## year-specific detection parameters

for(tt in 1:T){

# state=single

beta220[tt] ~ dnorm(mu220, tau220) # intercept

beta221[tt] ~ dnorm(mu221, tau221) # linear JD

beta222[tt] ~ dnorm(mu222, tau222) # quadratic JD

# state=pair

beta320[tt] ~ dnorm(mu320, tau320)

beta321[tt] ~ dnorm(mu321, tau321)

beta322[tt] ~ dnorm(mu322, tau322)

beta330[tt] ~ dnorm(mu330, tau330)

beta331[tt] ~ dnorm(mu331, tau331)

beta332[tt] ~ dnorm(mu332, tau332)

# state=repro

beta420[tt] ~ dnorm(mu420, tau420)

beta421[tt] ~ dnorm(mu421, tau421)

beta422[tt] ~ dnorm(mu422, tau422)

beta430[tt] ~ dnorm(mu430, tau430)

beta431[tt] ~ dnorm(mu431, tau431)

beta432[tt] ~ dnorm(mu432, tau432)

beta440[tt] ~ dnorm(mu440, tau440)

beta441[tt] ~ dnorm(mu441, tau441)

beta442[tt] ~ dnorm(mu442, tau442)

for(ii in 1:s){

for(jj in 1:k){

# state=single

logOR22[tt,ii,jj] <- beta220[tt] + beta221[tt]*Date[tt,ii,jj] + beta222[tt]*Date[tt,ii,jj]*Date[tt,ii,jj]

# state=pair/unrepro

logOR32[tt,ii,jj] <- beta320[tt] + beta321[tt]*Date[tt,ii,jj] + beta322[tt]*Date[tt,ii,jj]*Date[tt,ii,jj]

logOR33[tt,ii,jj] <- beta330[tt] + beta331[tt]*Date[tt,ii,jj] + beta332[tt]*Date[tt,ii,jj]*Date[tt,ii,jj]

# state=repro

logOR42[tt,ii,jj] <- beta420[tt] + beta421[tt]*Date[tt,ii,jj] + beta422[tt]*Date[tt,ii,jj]*Date[tt,ii,jj]

logOR43[tt,ii,jj] <- beta430[tt] + beta431[tt]*Date[tt,ii,jj] + beta432[tt]*Date[tt,ii,jj]*Date[tt,ii,jj]

logOR44[tt,ii,jj] <- beta440[tt] + beta441[tt]*Date[tt,ii,jj] + beta442[tt]*Date[tt,ii,jj]*Date[tt,ii,jj]

## populate the detection probability array

# state=unocc

p[tt,ii,jj,1,1] <- 1 # Detection Unocc if Unocc

p[tt,ii,jj,1,2] <- 0

p[tt,ii,jj,1,3] <- 0

p[tt,ii,jj,1,4] <- 0

# state=single

p[tt,ii,jj,2,1] <- 1/(1+exp(logOR22[tt,ii,jj])) # Detect Unocc if Single

p[tt,ii,jj,2,2] <- exp(logOR22[tt,ii,jj])/(1+exp(logOR22[tt,ii,jj])) # Detect Single if Single

p[tt,ii,jj,2,3] <- 0

p[tt,ii,jj,2,4] <- 0

# state=pair/non-repro

p[tt,ii,jj,3,1] <- 1/(1+exp(logOR33[tt,ii,jj])+exp(logOR32[tt,ii,jj])) # Detect Unocc if Pair

p[tt,ii,jj,3,2] <- exp(logOR32[tt,ii,jj])/(1+exp(logOR33[tt,ii,jj])+exp(logOR32[tt,ii,jj])) # Detect Single if Pair

p[tt,ii,jj,3,3] <- exp(logOR33[tt,ii,jj])/(1+exp(logOR33[tt,ii,jj])+exp(logOR32[tt,ii,jj])) # Detect Pair if Pair

p[tt,ii,jj,3,4] <- 0

# state=repro

p[tt,ii,jj,4,1] <- 1/(1+exp(logOR44[tt,ii,jj])+exp(logOR43[tt,ii,jj])+exp(logOR42[tt,ii,jj])) # Detect Unocc if Repro

p[tt,ii,jj,4,2] <- exp(logOR42[tt,ii,jj])/(1+exp(logOR44[tt,ii,jj])+exp(logOR43[tt,ii,jj])+exp(logOR42[tt,ii,jj])) # Detect Single if Repro

p[tt,ii,jj,4,3] <- exp(logOR43[tt,ii,jj])/(1+exp(logOR44[tt,ii,jj])+exp(logOR43[tt,ii,jj])+exp(logOR42[tt,ii,jj])) # Detect Pair if Repro

p[tt,ii,jj,4,4] <- exp(logOR44[tt,ii,jj])/(1+exp(logOR44[tt,ii,jj])+exp(logOR43[tt,ii,jj])+exp(logOR42[tt,ii,jj])) # Detect Repro if Repro

}

}

}

for (ii in 1:s) {

# impute occupancy

Occ[1,ii] ~ dcat(Phi[Block[ii],])

for (tt in 1:T-1) {

Occ[tt+1,ii] ~ dcat(TPM[Block[ii], tt, Occ[tt,ii], ])

}

# Likelihood

for (tt in 1:T) {

for (jj in 1:k) {

Detect[tt,ii,jj] ~ dcat(p[tt,ii,jj, Occ[tt,ii], ])

}

}

}

}
